# Liquid Chromatographic Strategies for Separation of Bioactive Compounds in Food Matrices

**DOI:** 10.3390/molecules23123091

**Published:** 2018-11-27

**Authors:** Chiara Cavaliere, Anna Laura Capriotti, Giorgia La Barbera, Carmela Maria Montone, Susy Piovesana, Aldo Laganà

**Affiliations:** Department of Chemistry, Sapienza University of Rome, Piazzale Aldo Moro, 00185 Roma, Italy; chiara.cavaliere@uniroma1.it (C.C.); giorgia.labarbera@uniroma1.it (G.L.B.); carmelamaria.montone@uniroma1.it (C.M.M.); susy.piovesana@uniroma1.it (S.P.); aldo.lagana@uniroma1.it (A.L.)

**Keywords:** bioactive compounds, carotenoids, liquid chromatography, peptides, polyphenols

## Abstract

Nowadays, there is an increasing attention for nutraceuticals and, in general, bioactive compounds naturally present in food. Indeed, the possibility of preserving human health and preventing disease (e.g., cardiovascular diseases, cancer etc.) by the intake of healthy food is attractive for both consumers and food industries. In turn, research in this field was also prompted significantly, with the aim of characterizing these bioactive compounds and ascribe to them a specific activity. The bioactive compounds can belong to several chemical classes. However, their chemical diversity and presence in complex matrices, such as food, make it challenging both their isolation and characterization. To tackle this issue, efficient separation systems are needed, which are mainly based on chromatography. In this context, this mini-review aims to provide the reader with an overview of the most relevant and recent approaches for the separation of the most common bioactive compounds in food, in particular polyphenols, phenols, carotenoids, and peptides, by liquid chromatography approaches.

## 1. Introduction

Recent years have witnessed an increased interest in nutraceuticals and bioactive compounds. Bioactive compounds are being studied in the prevention of cancer, heart disease, and other diseases (https://www.cancer.gov/publications/dictionaries/cancer-terms?cdrid=703278) [1]. The possibility to preserve health by consuming specific beneficial foods or to treat some diseases by consuming supplements containing natural components is attractive for both consumers and food industries. At lab-scale, efforts are aimed to extract new bioactive substances from food matrices and identify and assess their properties; at the scale-up level, more rapid, cheaper, and automatable approaches for recovering these high-value molecules are required.

Different separation techniques have been used for the investigation of bioactive compounds, which include high performance liquid chromatography (HPLC), gas chromatography (GC), but also other types of chromatographic approaches, such as thin layer chromatography which is an easy and low-cost approach especially employed for quality control of phytotherapeutics and plant drugs, the distribution of which was gradually overshadowed by the more powerful HPLC [2]. Supercritical fluid chromatography (SFC) and Ultra High Performance SFC [3,4,5] have also attracted considerable attention in the field of bioactive compounds research [6], especially for the investigation of carotenoids [7,8,9]. However, the techniques of choice are HPLC and GC [10,11], especially in their hyphenated version with tandem mass spectrometry (MS/MS) or high resolution MS for analyte characterization. Only when the substances are already known is it possible to use other detectors, such as the diode array detector (DAD).

Due to the complexity of food matrices and the great structural variety of the substances they contain, the separation of bioactive compounds and their analysis by HPLC is not an easy task, often requiring long run times. Nevertheless, in the last decades, as for other fields, research on bioactive compounds is obtaining advantages by the development of new HPLC stationary phase technologies, including sub-2 µm particles, partially porous particles, and porous monolithic polymers [12].

The employment of smaller-particle chromatographic packing materials allows improvement of resolution, efficiency, and method sensitivity, because the resulting chromatographic peaks are sharper and higher [12]. The high resulting backpressure can be alleviated by using short columns, which also guarantee low dead volumes. Moreover, the introduction of ultra (U)HPLC instruments, able to operate at pressure limits up to 1500 bar, equipped with increasingly efficient and reproducible thermostatting systems, allows operation with small internal diameter chromatographic columns (generally 2.1 mm i.d.) at higher flow-rate than classical HPLC instruments, thus resolving hundreds of compounds in very short run times.

A silica solid non-porous core coated with a thick porous layer constitutes the phases commercially known as fused-core, solid-core, porous-shell, and core-shell particles. Compared to sub-2 µm fully porous particles, partially porous particles have equivalent performance, but the advantage of lower backpressures, so that they can be even used with conventional HPLC systems [13].

Monolithic supports consist of a single rod of porous material with several unique features in terms of permeability and efficiency. These materials are prepared from either organic polymers (e.g., polymethacrylate, polyacrylamide, and poly(styrene-divinylbenzene)) or from inorganic polymers (e.g., silica, zirconia, titania, carbon) [14].

All these stationary phase technologies have advantages and drawbacks, and for deepening these topics, the reader can refer to the specific literature [14,15,16,17,18]. Nonetheless, separation efficiency is not only dependent on stationary phase typology, but it is strongly affected by the optimization of operational parameters, such as column temperature, mobile phase composition, and flow-rate [12]. In particular, because temperature affects both the kinetics and thermodynamics of the chromatographic process, operating at temperatures > 30 °C reduces solvent viscosity, leading to a lower column backpressure which can be explored to increase mobile phase flow-rate and speed up LC separations [12]. Generally, to avoid column deterioration, operating temperatures up to 50–60 °C are used.

Capillary and nano-LC, i.e., the miniaturized versions of conventional HPLC and UHPLC systems, have also been used in food analysis [19]. Nano-LC and capillary LC use chromatographic columns with i.d. up to 100 μm and flow rates of 50–800 nL min^−1^, and i.d. up to 320 μm and flow-rates of 1–100 μL min^−1^, respectively. The system miniaturization provides a big gain in sensitivity, particularly if separation is followed by electrospray ionization (ESI)-MS. Nonetheless, the employment of nano-LC is often limited to proteins and peptides analysis, since its applicability requires an extensive sample pretreatment and preconcentration, thus excluding the possibility of the direct crude extract analysis. Moreover, miniaturized LC could serve for identification of bioactive compounds, but is unsuitable for isolation of bioactive fractions of interest.

This review aims to provide the reader with an overview of the most relevant and recent approaches for the separation of bioactive compounds in food matrices by liquid chromatography techniques. Among the different compound classes investigated in bioactive compound analysis, special attention is given to the most common classes, especially phenols, polyphenols, carotenoids, and peptides.

## 2. Liquid Chromatography Modes

The basic principle of chromatographic separation is the different affinity of analytes for stationary and mobile phase. Various liquid chromatography modes are available, using different stationary phase typologies and chemistry, and the most used in the study of bioactive compounds are reversed-phase (RP), ion exchange (IEC), affinity, size-exclusion (SEC), and hydrophilic interaction liquid chromatography (HILIC). Most of these separation approaches are used in the final stage of analyte identification, however, application at the preparative stage is also possible, for reduction of matrix complexity, analyte purification, or complex mixture fractionation.

### 2.1. Reversed Phase Chromatography

The RP retention mechanism (adsorption and partition model) has been deeply investigated, and it is mainly based on hydrophobic interactions between the eluting molecules and the stationary phase [20]. Generally, either silica-bonded or polymeric-bonded octadecyl (C18), octyl (C8), or other alkyl stationary phases are used. Monomeric bonded stationary phases offer the highest separation efficiency, while polymeric stationary phases are more stable and resistant to operating conditions and in aqueous mobile phases, due to their cross-linked network [21]. Moreover, operating with the same mobile-phase composition, system properties are influenced by column packing morphology [22,23] and column length [24,25].

The RP C18 columns are the most used in various applications, including the study of bioactive compounds belonging to various chemical classes [26,27,28].

### 2.2. Ion Exchange Chromatography

IEC separation is mainly based on Coulombic interactions between charged groups of the analyte and the charged stationary phase [29], and it is widely employed for separating proteins and peptides. Indeed, IEC is often used as first dimension in multidimensional (MD)-LC in combination with RP chromatography, mainly in off-line mode, due to low compatibility of IEC mobile phase, which contains salts as peptide displacers, with the coupling to MS for detection, in order to avoid analyte signal suppression. Nevertheless, elution can also be performed by varying the pH value of the mobile phase. For protein and peptide separation, strong cation exchange chromatography is generally employed, however anion exchange chromatography finds applications, too.

### 2.3. Hydrophilic Interaction Liquid Chromatography

HILIC consists in a liquid chromatographic technique where polar or ionized compounds can be separated on a polar stationary phase with polar solvents containing water as a minor constituent of the mobile phase [30]. Among HILIC mechanism hypotheses, the most accredited one was proposed by Alpert [31], involving partitioning of solutes between the bulk mobile phase and a layer enriched with water and partially immobilized on the stationary phase surface. However, the actual separation mechanism is more complex, because differences in the chromatographic selectivity of various polar compounds indicate that adsorption on the polar centers on the solid phase surface may also contribute to the retention [32].

The main advantage of HILIC over classical RP mode is its ability in retaining strongly and moderately polar and ionic compounds. Indeed, RP and HILIC possess a very different selectivity and their orthogonality has been used in comprehensive LC × LC applications [30]. Recently, HILIC has found several applications in the analysis of food compounds [33].

### 2.4. Affinity Chromatography

Affinity chromatography exploits a biologically-related agent immobilized on a stationary phase to purify or analyze specific sample components. There are various modes to achieve it, the main ones being bioaffinity chromatography, immunoaffinity chromatography, dye-ligand, and biomimetic affinity chromatography, immobilized metal-ion affinity chromatography, and analytical affinity chromatography [34].

### 2.5. Countercurrent Chromatography

Countercurrent chromatography (CCC) is a chromatographic separation and purification technique based on the liquid-liquid partition mechanism [35]. It comprises all the LC forms using two immiscible liquid phases without any solid support [36]. The liquid stationary phase is held in place by centrifugal force, while the immiscible mobile phase is pumped through it. Multiphasic solvent systems can be used with various elution modes. Because CCC uses a liquid stationary phase without any solid support, the main advantages over other conventional LC approaches are the high selectivity, thanks to a variety of biphasic solvent systems, the high loading capacity, the possibility of direct application of crude extracts with no sample pretreatment due tolerance of particulate matter, the possibility of total recovery of the injected sample, the absence of irreversible solute adsorption, low risk of sample denaturation, and easy scale-up [36,37]; furthermore, if the analytes are not pH-sensitive, there are no limits in pH choice [38]. The disadvantages over LC techniques with a solid support include the limited distribution of CCC in the scientific community, the time-consuming optimization, the risk of instability of the solvent system and creation of emulsions [37]. CCC is especially suitable for isolation of bioactive compounds, typically on preparative and semipreparative scale (sample amounts are 100 mg–10 g). In this regard, compared to LC techniques with a solid support, the resolution can be lower [37]. Nevertheless, this technique can be coupled with other on-line separation techniques [35], also in MD-CCC mode, by means of switching valves to interface the columns or different detectors, including NMR and MS [36]. Moreover, in the literature there are several applications of high-speed (HS)CCC for the purification/separation of various bioactive chemical classes from plants [36,38,39,40].

## 3. Multidimensional Chromatography

Food matrices are very complex and, in most cases, the compounds of interest are minor components. Therefore, a single column chromatographic process does not often provide the required resolving power and more powerful separation tools are demanded [10]. Indeed, even when coupled to MS, LC cannot be selective enough if many target compounds are structurally similar or isomers. In such cases, MD-LC could be able to resolve sample complexity. It is based on the combination of two or more orthogonal separation steps, obtaining a peak capacity and a resolving power higher than any improved one-dimensional method [10,41] when columns with different selectivities are used in the two dimensions. The total peak capacity of the fully orthogonal two-dimensional chromatography system can be theoretically estimated as a product of the peak capacities in the first and second dimension [42]. Even if no orthogonality can be obtained, some works exploit RP × RP under the same pH condition for MD-LC to fractionate bioactive compounds, especially peptides, in order to reduce the number of possible candidates responsible of a tested bioactivity [43,44].

MD-LC can be performed either in heart-cutting (LC-LC) and comprehensive LC (LC × LC) modes, in both off-line or on-line fashion. While in comprehensive LC × LC the entire eluate from the first column is transferred to the second column and analyzed with very fast gradients (because run time for the second dimension has to match the collection time of the first dimension effluent), in heart-cutting LC-LC, only one or more fractions of the first dimension effluent are injected onto the second dimension column; in this case, a peak from the first dimension is sampled as a whole and then analyzed by a gradient with a longer run time than the collection time. Due to the more technical complexity of the on-line version, often the first dimension is carried out off-line, with the advantage that each dimension can be independently optimized.

## 4. Analysis of Bioactive Compounds

Among non-peptide bioactive compounds, phenols are the most abundant and investigated ones in plant food. Within phenols, many compounds have the same molecular formula or are positional isomers, therefore for a correct identification, it is important to obtain a suitable chromatographic separation. For example, flavonoids can be hydroxylated in various positions of their carbon backbone, and one or more of these hydroxyl groups can be methylated, acetylated, or prenylated. Moreover, flavonoids can occur in both free and glycosylated forms (as *O*- or *C*-glycosides) with different sugar moieties, which can be further substituted by acyl residues such as malonyl and acetyl [45]. For the separation of these natural compounds, C18 is the most used stationary phase, generally in UHPLC fashion (Table 1).

Other molecules, which are often investigated for their high antioxidant activity, are the lipid-soluble carotenoids, characterized by a long chain of alternating double and single bonds. They can be found in both plant and animal food [7,8,9].

### 4.1. Separation of Bioactive Compounds by One-Dimensional Chromatography

Generally, in food samples, small bioactive molecules are determined by one-dimensional separation.

When the goal is the untargeted profiling of a class of bioactive compounds in a plant matrix, an efficient separation system is necessary to avoid phenomena, such as signal suppression, and it is especially needed in the presence of isomeric metabolites, which are otherwise indistinguishable. To characterize the high polar glucosinolates in cauliflower, Capriotti et al. [25] compared three RP chromatographic columns with different stationary phases, column configuration, particle size, and mobile phases. The largest number of identified ions was obtained with two serially connected C18 columns, indicating how column length appears fundamental to resolve complex samples, as a longer column set-up improves isomeric analyte identification.

Some authors proposed a bioassay guided fractionation (BGF) strategy to find out the target bioactive candidates by repeated fractionations and isolations [48]. However, the main drawbacks of BGF are the long time required for the various steps, which could lead to active compound degradation and the loss of information about the synergistic effects with the other compounds. To optimize the process, a totally “data-driven” method (i.e., without needing a prior knowledge about the mixture), which combined “Sure Independence Screening” (SIS) and “interval Partial Least Squares” (iPLS), was developed: while SIS can quickly and accurately locate the potential bioactive components in a small range, iPLS further eliminates uninformative variables.

### 4.2. Separation of Bioactive Compounds by Counter Current Chromatography

Generally, crude plant extracts are too complex for a direct analysis by HPLC [38], but they can be analyzed by CCC, instead.

When developing a CCC method, the choice of the solvent system is of outmost importance to obtain the suitable isolation/separation of the target molecules from plant extract. Therefore, some authors also proposed multiple-solvent systems in which the polarities vary largely depending on the ratio of each solvent in the system. Das Neves Costa and Guimarães Leitão [45] observed that in the literature free flavonoids were frequently fractionated with the hexane–ethyl acetate–methanol–water solvent system.

The CCC technique is often used to isolate bioactive constituents of medicinal plants. As alternative to time-consuming traditional silica-gel and macroporous resin column chromatography, already used for the separation of diverse components of *Salvia plebeia*, the combination of three different CCC elution modes, namely classical CCC, elution–extrusion CCC, and recycling CCC, in a two-step HSCCC method, allowed the separation of nine major flavonoids and caffeic acid derivatives from this plant matrix [49].

One-dimensional CCC provides initial purification, but to improve bioactive peak resolution, MD-CCC strategies are often required. In order to improve CCC separation efficiency, it is possible to increase the column length by using the closed-loop recycling elution mode. Han et al. [50] used a bioactivity-guided cut CCC separation strategy to isolate target bioactive molecules from *Scutellaria baicalensis Georgi*: the fractions, with assessed bioactivity, were either collected or further purified by closed-loop recycling elution mode. The main advantage is that the entire CCC separation can be performed on a single apparatus.

Very interesting is the possibility of coupling HSCCC with MS via ESI or atmospheric pressure chemical ionization (APCI) sources [36]. For example, Gutzeit et al. in 2007 described a preparative HSCCC/ESI-MS^*n*^ setup with automatic fraction collection for target-guided fractionation and isolation of polyphenols from *Hippophaë rhamnoides* juice [51]. A preparative HSCCC/ESI-MS^*n*^ setup was also used for separation and online identification of coumarins from *Peucedanum praeruptorum Dunn* [52]. The two-phase solvent system was constituted by the light petroleum–ethyl acetate–methanol–water solvent system, and ammonium formate was added into the mobile phase to increase the ionization efficiency.

*Hericium erinaceum* is an edible fungus rich in bioactive compounds such as polysaccharides, pyrones, sterols, terpenoids, phenols, etc. However, little was known about flavonoids, which were studied by separation and purification using a HSCCC method with a two-phase solvent system, namely chloroform–dichloromethane–methanol–water. With the aid of different spectroscopic and spectrometric techniques, including ESI-MS, He et al. [53] identified daidzein and genistein in the fungus mycelium.

### 4.3. Separation of Bioactive Compounds by Multidimensional Chromatography

In the last years, RP-LC×RP-LC [54] and HILIC×RP-LC [55,56,57] are the most used combinations for phenols analysis [10]. In fact, phenols comprise various chemical classes, such as phenolic acids, lignans, flavonoids (further subdivided in flavones, isoflavones, flavones, anthocyanins, etc.), therefore their determination often requires integrated analytical strategies.

Lignans with a dibenzocyclooctadiene skeleton are considered the bioactive compounds of the traditional Chinese medicine *Schisandra chinensis*. The structures of these bioactive compounds are very similar, thus for their chromatographic separation Wang et al. [42] compared four on-line MD-LC set ups (four stationary phases, i.e., cyano, phenyl, C8, and immobilized liposome, in the first dimension and monolithic C18 for the second dimension). The best orthogonality was obtained with the immobilized liposome chromatography column (able to mimic the biological membranes), which allowed separation of more than 40 compounds.

Carotenoids pose analytical challenges similar the ones previously discussed for phenols. This complex class of compounds is generally separated by C30 columns in one-dimensional chromatography. To improve resolution, MD-LC is the technique of choice for investigation of carotenoids in complex real-world matrices, such as red mamey (*Pouteria sapote*) fruit [58]. In this work, a cyano microbore column was exploited for the first dimension and separation of analytes by NP into groups based on analyte polarity. In the second dimension, separation was performed on a fused-core RP column to separate analytes based on their hydrophobicity. The use of a microbore column in the first dimension allowed minimizing the possible problems due to solvent incompatibility between NP and RP. In other MD-LC applications, the NP cyano stationary phase in the first dimension was coupled with RP monolithic C18 stationary phase in the second dimension [59,60].

Other classes of compounds commonly found in food have been separated by MD-LC, for example, triacylglycerols and phospholipids. We invite the reader to refer to the review by Cacciola et al. for further reading [10].

## 5. Analysis of Protein-Derived Bioactive Peptides

Several bioactivities have been ascribed to protein-derived bioactive peptides [68], including antioxidant, antihypertensive, and antimicrobial properties [69]. Generally, bioactive peptides are protein fragments from 2 up to 40 amino acids long, whose activity is displayed only after hydrolysis of the protein they are encrypted in. The peptide release may occur during gastrointestinal digestion by proteolytic enzymes or during food processing (ripening, fermentation, cooking) or storage [70]. Milk [61,71], egg, meat, and fish [62] are the most investigated sources of bioactive peptides, but some plant products [63] (e.g., cereals, legumes, seeds, etc.) and more recently algae [64] are also of interest.

In their recent review, Piovesana et al. [70] evidenced that in several works the issue of the identification of single bioactive peptides is neglected, since the goal is only the isolation of a bioactive fraction or extract to use for supplement preparation. Nonetheless, isolation and purification of bioactive peptides are also very important to evaluate the bioactivity properties by in vitro and in vivo assays [72]. HPLC is the most powerful technique to isolate and purify bioactive peptides, and performance of RP, IEC, HILIC, size exclusion, and affinity chromatography have been all tested for this aim; however, a better separation of peptides requires the combination of more chromatographic techniques [72]. Indeed, generally, these bioactive peptides are low-abundance ones; therefore, by using MS for detection, their signal could be suppressed by more abundant species.

### 5.1. Separation of Bioactive Peptides by One-Dimensional Chromatography

Generally, if peptide separation is limited to one-dimension, C18 is the stationary phase of choice. However, HILIC can be more suitable for small peptides that are scarcely retained on conventional RP phases, as described by Vásquez-Villanueva et al. [65]. In fact, they employed both RP and HILIC chromatographic separation to obtain the identification of most hydrophobic as well as less hydrophobic peptides from peach seed hydrolysates. From LC-MS/MS analyses, it appeared that the RP-LC chromatogram was richer than the HILIC one.

### 5.2. Separation of Bioactive Peptides by Multidimensional Chromatography

#### 5.2.1. Off-Line Separation

Egg is a recognized source of bioactive substances, which could be extracted at industrial scale. However, to better valorize the extractable peptides at industrial scale, an enzymatic hydrolysis by a non-commercially available serine proteinase of *Cucurbita ficifolia* was applied to protein extract [46]. Then, to isolate the peptides of interest with antihypertensive (angiotensin converting enzyme (ACE) inhibitor) activity, sequential separation/fraction steps were employed. After an ultrafiltration (molecular weight cut-off of 30 kDa) step, six fractions were obtained by gel filtration chromatography on a Zorbax GF-250 column at 30 °C. Four fractions showed ACE-inhibitor activity, and the most active one was further purified by RP C18 column obtaining seven major fractions. The choice of a non-commercially available enzyme seems to be the key factor in the valorization of egg white as a good source of antihypertensive peptides, however this fact could also limit the application of the method at lab-scale. Very likely, the initial step of membrane ultrafiltration for removing non-hydrolyzed proteins and residue protease could be avoided by optimizing hydrolysis conditions.

Lunasin is a 43 amino acid-long peptide with several ascribed bioactivities, from anti-inflammatory to anticancer. For its isolation from soybean, an isoelectrofocusing fractionation of protein extract was carried out before RP LC-MS analysis [47]. In this case, the combination of two orthogonal separation steps was necessary to reduce the signal suppression typical of ESI matrix effect and detect a single target peptide. Three other tested strategies, namely matrix dilution, and the employment of two alternative RP chromatographic columns (either a polar RP C18 column and a 2000 Å-perfusion column), as well as different SPE cartridges, were ineffective in enhancing the lunasin signal.

The same peptide, lunasin, was isolated form quinoa extract in different steps: the ultrafiltrated fraction (1 kDa < MW < 10 kDa) was further purified using Q-Sepharose fast flow chromatography and analyzed or again further purified by diethylaminoethyl IEC [73].

Zenezini Chiozzi et al. [66] evaluated the possibility of recovering bioactive peptides from cauliflower by-products (leaves and stems). After hydrolysis of protein extract, the purification of peptides was carried out by MD-LC employing RP and HILIC. Only the fractions with an assessed bioactivity were characterized by nanoLC-MS/MS and identified. After bioinformatic in silico analysis confirmation and the synthesis of putative bioactive peptides, three novel ACE-inhibitory peptides were successfully identified and validated. In this case, trypsin and a mixture of pepsin and pancreatin were used as proteolytic enzymes. The same group, in view of a scale-up strategy, changed the proteolytic enzyme, separation, and purification strategies [67]. In particular, the cheaper enzyme Alcalase was chosen, and the sample was first fractionated by preparative C18 RP-HPLC, then the most active fractions were analyzed by nanoLC-ESI-MS/MS using a C18 UHPLC column to identify the peptides of interest.

In MD-LC, orthogonality can be obtained by varying mobile phase pH condition in RP couplings [64], as exploited for the bioactive peptide investigation of *Tetradesmus obliquus* microalgae protein digest, where separation was performed first at low pH (by trifluoroacetic acid), then at high pH (ca. 10 by ammonium formate).

Bioactive peptides could also be endogenous in the food matrix; therefore, no enzymatic hydrolysis is required. For instance, in the investigation of donkey milk bioactive peptides, RP and HILIC were combined in an off-line MD-LC fractionations system [61]. Only the fractions showing a significant ACE-inhibitory or antioxidant activity were subject to the second-dimension separation, and further analyzed by nanoRP-LC-MS/MS. This work was an evolution of a previous one by the same research group [74], where the naturally occurring peptides were identified and the bioactivities tested in the entire extract without any further isolation step.

#### 5.2.2. On-Line Separation

For MD-LC on-line separation of food peptides, we invite the reader to refer to the review by Cacciola et al. [10]. RP-LC in both dimensions could be chosen to overcome the use of salts in the first dimension, which are required for IEC [75]. The combination RP at high pH (9) × RP at low pH (2) was successfully tested to separate peptides of the soluble fraction of milk generated during the fermentation process (four weeks from the expiration date) [76]. Despite the use of the same separation method (RP) in both dimensions, this online comprehensive LC × UHPLC platform obtained high peak capacity values and satisfactory selectivity.

## 6. Conclusions

Separation of bioactive compounds represents a difficult task, especially when the compounds of interest belong to different chemical classes. When the analysis is aimed to a single chemical class, not only the choice of the optimal stationary phase, but also of the best operating conditions is of outmost importance. To obtain the best separation efficiency, the employment of a MD-LC technique, either on-line and off-line, is recommended. The MD-LC approach is now well consolidated in the analysis of bioactive peptides, whereas in the study of small molecules it is less applied [77]. Finally, besides an efficient chromatographic separation, the employment of MS, particularly high-resolution MS, appears mandatory to achieve the correct identification of bioactive compounds in the complexity of a food extract.

## Figures and Tables

**Table 1:** List of bioactive compounds with related natural source, separation conditions, and detection used in the research papers discussed in the mini review. For comparative works, only the best conditions were reported. The following abbreviations were used: acetonitrile (ACN); angiotensin-converting enzyme (ACE); formic acid (FA); high-speed counter-current chromatography (HSCCC); matrix-assisted laser desorption ionization (MALDI); methanol (MeOH); “Sure Independence Screening” “interval Partial Least Squares” (SIS–iPLS); time of flight (TOF); trifluoroacetic acid (TFA).

Sample	Compounds	Separation Conditions	Detector	Results	Ref.
*Fragaria × ananassa*	flavonoids, phenolic acids, dihydrochalcones, ellagitannins, proanthocyanidins	RP: core–shell C18 column (100 mm × 2.1 mm, 2.6 μm); 40 °C, water/ACN both with 0.1% FA (*v*/*v*), 600 μL min^−1^	hybrid quadrupole-Orbitrap	113 compounds tentatively identified, 18 compounds identified	[23]
*Fragaria × ananassa*	anthocyanins, dihydrochalcones, dihydroflavonols, dihydroflavanols, flavanones, flavan-3-ols, proanthocyanidins, ellagitannins	2 × C18 columns, (100 × 2.1 mm, 2.6 μm) 40 °C, water/ACN both with 0.1% FA (*v*/*v*), 600 μL min^−1^	hybrid quadrupole-Orbitrap	89 compounds tentatively identified	[24]
*Brassica oleracea L. var. botrytis*	glucosinolates	2 in series C18 columns, (100 × 2.1 mm, 2.6 μm) 40 °C, water/ACN both with 0.1% FA (*v*/*v*), 600 μL min^−1^	hybrid quadrupole-Orbitrap	51 compounds tentatively identified	[25]
*Phaseolus vulgaris*	soyasaponins	C18 column (150 × 4.6 mm, 2.6 μm), water/ACN both with 0.1% FA (*v*/*v*), 800 μL min^−1^	Fourier transform ion cyclotron resonance MS + infrared multiphoton dissociation	8 compounds	[26]
*Annona cherimola* Mill.	sugars, amino acids, phenolic acids and derivatives, flavonoids, phenylpropanoids, other polar compounds	C18 column (4.6 mm × 100 mm, 2.7 μm), 25 °C, water with 1% acetic acid (*v*/*v*, phase A)/ACN (phase B), 0.8 mL min^−1^	TOF MS	77 compounds	[27]
*Vaccinium myrtillus* L.	anthocyanins	C18 (150 × 4.6 mm, 5 μm), 25 °C, (A) water/FA (9:1, *v*/*v*, phase A), MeOH/water/FA (5:4:1, *v*/*v*/*v*, phase B), 1 mL min^−1^	DAD, ESI-MS	14 compounds	[28]
commercial crude β-sitosterol standard (purity ∼60%)	phytosterols	HSCCC, solvent system: *n*-hexane/MeOH/aqueous silver nitrate solution (34:24:1, *v*/*v*/*v*), 1000 rpm, 1 mL min^−1^	GC-MS	sitostanol (>99%) and β-sitosterol (∼99%), mixture of campesterol and stigmasterol	[39]
*Schisandra chinensis*	lignans	on-line MD-LC (1) immobilized liposome chromatography (200 mm × 4.6 mm, 5µm); 10 mmol L^−1^ ammonium acetate (pH 6.8), 1.0 mL min^−1^, 25 °C. (2) monolithic C18 (100 mm × 4.6 mm), water/ACN, 3.0 mL min^−1^.	DAD, ESI-MS	>40 compounds separated, 14 identified	[42]
*Palmaria palmata*	Antidiabetic (dipeptidyl peptidase IV inhibitor) peptides	Off-line MD-LC (1) C18 semi-preparative column (250 × 15 mm, 10 μm) water/ACN both with 0.1% FA, 5 mL min^−1^ (2) C18 column (2.1 × 50 mm, 1.7 μm) water/ACN both with 0.1% FA, 0.2 mL min^−1^	ESI-MS	3 bioactive peptides	[43]
Sweet potato	antioxidant peptides	Off-line MD-LC (1) C18 column (21.2 × 150 mm), water/ACN both with 0.1% TFA, 10 mL min^−1^ (2) C18 column (75 μm × 150 mm; 5 μm), water/ACN both with 0.1% FA, 300 nL min^−1^	LTQ linear ion trap	5 peptides	[44]
Egg	ACE-inhibitor peptides	Off-line MD-LC (1) gel filtration chromatography GF-250 (250 mm × 4.6 mm); 0.02 mol L^−1^ phosphate buffer (pH 7.2) with 0.2% NaCl, 30 °C, 0.5 mL min^−1^. (2) C18 column (50 mm × 1.8 mm), water/ACN with both 0.1% TFA, 1 mL min^−1^, 30 °C.	DAD; MALDI TOF/TOF	113 compounds tentatively identified, 18 compounds identified	[46]
Soybean	Lunasin	(1) isoelectrofocusing (2) C18 column (100 mm × 2.1 mm, 2.7μm), water/ACN both with 0.3% (*v*/*v*) acetic acid, 0.4 mL min^−1^, 30 °C	ESI-QTOF-MS	Lunasin	[47]
*Radix Puerariae Lobatae*	main bioactive signatures	C18 column (150 × 4.6 mm, 5 μm), water/MeOH both with 0.1% phosphoric acid or ACN/0.3% acetic acid	DAD, SIS–iPLS	9 main bioactive signatures	[48]
*Salvia plebeia* R.Br.	polyphenols	two-step HSCCC, three solvent systems: *n*-hexane/ethyl acetate/ethanol/water (4:6.5:3:7, *v*/*v*), methyl tert-butyl ether/ethyl acetate/*n*-butanol/MeOH/water (6:4:1:2:8, *v*/*v*), *n*-hexane/ethyl acetate/MeOH/water (5:5.5:5:5, *v*/*v*)	HPLC-DAD	9 compounds	[49]
*Scutellaria baicalensis* Georgi	lysine-specific demethylase 1 inhibitors	HSCCC, solvent system: ethyl acetate/MeOH/water	HPLC-DAD	6 natural lysine-specific demethylase 1 inhibitors	[50]
*Hippophaë rhamnoides* L. ssp. *rhamnoides*	polyphenols	MD-HSCCC, solvent system: *n*-hexane/*n*-butanol/water (1:1:2, *v*/*v*/*v*)	MS (direct coupling)	5 compounds	[51]
*Peucedanum praeruptorum* Dunn	coumarins	HSCCC, solvent system: light petroleum/ethyl acetate/MeOH/water (5:5:6:4, *v*/*v*)	MS (direct coupling)	7 compounds	[52]
*Hericium erinaceum*	isoflavones	HSCCC, solvent system: chloroform/dichloromethane/MeOH/water (4:2:3:2, *v*/*v*/*v*/*v*)	Infrared spectroscopy, MS, nuclear magnetic resonance	2 compounds	[53]
*Fructus aurantii*	polymethoxylated flavones	Off-line MD-LC (1) C8 column (250 mm × 4.6 mm, 5 μm), 30 °C, water/MeOH, 0.25 mL min^−1^ (2) C18 column (250 mm × 4.6 mm, 5 μm), 30 °C, water/MeOH, 0.25 mL min^−1^	DAD, ESI-MS	42 compounds tentatively identified	[54]
Cocoa	procyanidins	Off-line, stop-flow or on-line MD-LC (1) HILIC Diol-100 column (250 mm × 1 mm, 5 μm), ACN/acetic acid (99:1, *v*/*v*, phase A), MeOH/water/acetic acid (94.05:4.95:1, *v*/*v*/*v*, phase B) (2) C18 column (50 mm × 4.6 mm, 1.8 μm), water/ACN both with 0.1% FA	DAD	Comparative study	[55,56]
Cocoa, red grape seed and green tea	phenolics	On-line MD-LC (1) HILIC Diol-100 column (250 mm × 1 mm, 5 μm), ACN/acetic acid (99:1, *v*/*v*, phase A), MeOH/water/acetic acid (94.05:4.95:1, *v*/*v*/*v*, phase B), 25 μL min^−1^ (2) C18 column (50 mm × 4.6 mm, 2.6 μm), 0.1% FA/acetonitrile, 1.5 mL min^−1^	DAD, ESI-MS	Comparative study	[57]
*Pouteria sapote*	carotenoids	On-line MD-LC (1) Cyano column (250 × 1.0 mm I.D., 5-μm), *n*-hexane/butyl acetate/acetone (80:15:5, *v*/*v*/*v*, phase B), *n*-hexane (phase A), 10 μL min^−1^ (2) C18 column (50 × 4.6 mm, 2.7 μm), 2-propanol (phase A), water/ACN (90:10, *v*/*v*, phase B), 3 mL min^−1^	DAD, MS	23 compounds identified	[58]
Red Orange Essential Oil	carotenoids	On-line MD-LC (1) Cyano column (250 × 1.0 mm, 5-μm), *n*-hexane/butyl acetate/acetone (80:15:5, *v*/*v*/*v*, phase B), *n*-hexane (phase A), 10 μL min^−1^ (2) C18 column (100 × 4.6 mm, 2.7 μm), 2-propanol (phase A), water/ACN (20:80, *v*/*v*, phase B), 5 mL min^−1^	DAD, MS	37 compounds	[59]
Red chili peppers	carotenoids	On-line MD-LC (1) Cyano column (250 × 1.0 mm, 5-μm), *n*-hexane/butyl acetate/acetone (80:15:5, *v*/*v*/*v*, phase B), *n*-hexane (phase A), 10 μL min^−1^ (2) 2 × C18 columns (30 × 4.6 mm, 2.7 μm), 2-propanol (phase A), water/ACN (20:80, *v*/*v*, phase B), 5 mL min^−1^	DAD, ion trap-TOF	33 compounds	[60]
Donkey milk	antioxidant and ACE-inhibitor peptides	Off-line MD-LC (1) polymeric RP (4.6 × 250 mm, 5 μm), water/ACN both with 0.1% FA; 1 mL min^−1^ (2) HILIC column (100 × 2.1 mm, 2.6 μm), ACN/5 mmol L^−1^ HCOONH_4_ (50:50, *v*/*v*, phase B), ACN/5 mmol L^−1^ HCOONH_4_ (90:10, *v*/*v*, phase A); 300 μL min^−1^ (3) C18 (500 × 0.75 mm, 2 μm), water/ACN both with 0.1% FA, 200 µL min^−1^	DAD, hybrid Orbitrap	2 antioxidant peptides, 2 ACE-inhibitor peptides	[61]
*Dicentrarchus labrax*	Known bioactive peptides	C18 column (500 × 0.75 mm, 2 μm) water/ACN both with 0.1% FA, 200 µL min^−1^	hybrid Orbitrap	44 peptides antimicrobial peptides	[62]
Soybean seeds and milk	Known bioactive peptides	C18 column (500 × 0.75 mm, 2 μm) water/ACN both with 0.1% FA, 200 µL min^−1^	hybrid Orbitrap	24 antimicrobial peptides	[63]
*Tetradesmus obliquus* microalgae	ACE-inhibitor and antioxidant peptides	Off-line MD-LC (1) polymeric C18 (19 × 250 mm, 5 μm), water/MeOH both with 0.1% TFA, 17 mL min^−1^ (2) C18 column (10 × 250 mm, 5 μm), 10 mmol L^−1^ HCOONH_4_ (pH 10, phase A), MeOH/water (90:10, *v*/*v*, with 10 mmol L^−1^ HCOONH_4_, pH 10), 7 mL min^−1^ (3) C18 column (500 × 0.75 mm, 2 μm), water/ACN both with 0.1% FA, 200 µL min^−1^	DAD, hybrid Orbitrap	4 ACE-inhibitor peptides	[64]
Peach byproducts	antioxidant peptides	Off-line MD-LC (1) C18 column (100 mm × 2.1 mm, 2.7 μm), 0.3% (*v*/*v*) acetic acid (phase A), ACN with 0.3% (*v*/*v*) acetic acid (phase B), 0.3 mL min^−1^ (2) silica HILIC (100 × 2.1 mm, 2.7 μm), ACN with 65 mmol L^−1^ ammonium acetate (phase A), 65 mmol L^−1^ ammonium acetate (phase B), 0.3 mL min^−1^	Q-TOF	18 peptides	[65]
Cauliflower byproducts	ACE-inhibitor peptides	(1) polymeric C18 column (19 × 250 mm, 5 μm), water/ACN both with 0.1% FA, 1 mL min^−1^ (2) HILIC (100 × 2.1 mm, 2.6 μm), ACN/5 mmol L^−1^ HCOONH_4_ (50:50, *v*/*v*; phase B), ACN/5 mmol L^−1^ HCOONH_4_ (90:10, *v*/*v*, phase A), 300 µL min^−1^ (3) C18 column (25 × 0.75 mm, 2.2 μm), water/ACN both with 0.1% FA, 250 µL min^−1^	DAD, hybrid Orbitrap	3 ACE-inhibitor peptides	[66]
Cauliflower by-products	antioxidant and ACE-inhibitor peptides	Off-line MD-LC (1) polymeric C18 column (19 × 250 mm, 5 μm), water/MeOH both with 0.1% TFA, 17 mL min^−1^ (2) C18 column (25 × 0.75 mm, 2.2 μm), water/ACN both with 0.1% FA, 250 µL min^−1^	DAD, hybrid Orbitrap	4 peptides	[67]
